# Mitochondria in Ageing and Diseases: Partie Deux

**DOI:** 10.3390/ijms241210359

**Published:** 2023-06-20

**Authors:** Hannah Tobias-Wallingford, Giuseppe Coppotelli, Jaime M. Ross

**Affiliations:** 1 George and Anne Ryan Institute for Neuroscience, University of Rhode Island, Kingston, RI 02881, USA; 2 Department of Biomedical and Pharmaceutical Sciences, College of Pharmacy, University of Rhode Island, Kingston, RI 02881, USA

The past several decades has seen a huge expansion of the knowledge and research of mitochondrial dysfunction and the role it plays in ageing and age-related diseases. Mitochondrial dysfunction can be caused by mutations or deletions in nuclear and/or mitochondrial DNA (mtDNA). Additionally, the breakdown of quality control mechanisms can drive this dysfunction. Mitochondria play critical roles in not only energy production but calcium buffering and homeostasis, steroid synthesis, cell growth, apoptosis, inflammation, and reactive oxygen species production. Key signs of mitochondrial dysfunction include decreased ATP production, decreased mitochondrial membrane potential, swollen mitochondria, damaged cristae, increased oxidative stress, and decreased mtDNA copy number.

Mitochondrial dysfunction has massive impacts on overall health and longevity. Specifically, mitochondrial dysfunction is implicated in ageing, as well as many disease states such as neurodegenerative diseases, including Alzheimer’s disease and Parkinson’s disease, and metabolic disorders, cancers, and inflammatory disorders. Understanding the implications of mitochondrial dysfunction, including what drives it, its long-term impacts, and ways to mediate it, is crucial for improving health and quality of life of the aged population (defined as persons >65 years of age) as their proportion continues to increase.

The purpose of this Special Issue, “Mitochondrial Dysfunction in Ageing and Diseases: Partie Deux”, published in the *International Journal of Molecular Sciences* [1], is to understand the current progress and standing of research on mitochondrial dysfunction through reviews and original research. A total of five papers, including one article and four reviews, have been published as part of the Special Issue as detailed in Table 1. Research focuses ranging from mitochondrial DNA mutations, redox signaling, and mitochondrial nutrients to liver function, cognition, and mineralocorticoid receptors are included.

Geary [2] provides an important overview with a review of the intertangling of cell biology and cognitive health with mitochondrial dysfunction at a critical intersection. This work highlights the importance of mitochondrial energy production for cognitive abilities, with brain network functioning therefore being important in overall biological fitness. Energy is essential for complex biological networks including cognitive abilities, and hence functional mitochondria are critically important for these processes. With additional studies, this information has the potential to help find biomarkers for earlier diagnosis of a variety of diseases, such as Alzheimer’s disease.

A study by Lefranc et al. [3] reviews mitochondrial function more specifically by examining the relationship between mitochondria and adipocyte-mineralocorticoid receptors (MRs). Additionally, Dabravolski et al. [4] examines non-alcoholic fatty liver disease (NAFLD) through the lens of mitochondrial function. The work on MR expression and mitochondrial quality control and function by Lefranc et al. [2] is the only research article in this Special Issue, and shows that MR expression is increased in adipose tissue with obesity. This work describes findings using a transgenic mouse model that overexpresses MR that presents with an increase in mitochondrial oxygen species, as well as an alteration in mitochondrial function. The mitochondria balance was found to be shifted towards fission and inhibiting fusion processes. Therefore, the quality control mechanisms of mitochondria appeared to be impaired with MR overexpression. This work suggests the potential of MR antagonism as a treatment to regulate mitochondria. Non-alcoholic fatty liver disease (NAFLD) is discussed through the lens of being a mitochondrial disease by Dabravolski et al. [4]. NAFLD is very common and linked with other disease states. Considering the role of mtDNA mutations in NAFLD is important given the impact of mitophagy, reactive oxygen species, lipotoxic byproducts of beta-oxidation, and mtDNA in the progression and development of NAFLD.

Another aspect of age-related decline that is important to consider when understanding the impacts of mitochondrial function is sarcopenia, a decline in muscle mass and function. Foreman et al. [5] reviews the impact of reactive oxygen species on the development of sarcopenia due to dysregulation of redox signaling. Although the primary driver is still unclear, the interplay between mitochondrial homeostasis and peroxiredoxins in redox signaling may be critical in understanding sarcopenia in ageing.

Given the important points brought together by the other articles in this Special Issue, Pagano et al. [6] bring forward a review on potential strategies to protect mitochondria with mitochondrial nutrient mixes. Mitochondrial cofactors or nutrients including α-lipoic acid (ALA), which is important in the citric acid cycle, Coenzyme Q10 (CoQ10), which is involved in the electron transport chain, and carnitine (CARN), the main carrier of acyl groups across the mitochondrial membrane, have been studied for their possible therapeutic effects in “mitochondrial medicine.” Clinical trials have not studied the possibility of administering all three of these mitochondrial nutrients together. The lack of knowledge in this area, as the authors point out, needs to be further rectified given the potential for therapeutic benefits in a variety of mitochondrial dysfunction-related disorders.

**Table 1:** Summary of papers in the Special Issue.

	Title	Topics/Keywords	Type
Dabravolski et al. [4]	Mitochondrial Mutations and Genetic Factors Determining NAFLD Risk	NAFLD; NASH; chronic inflammation; fibrosis; mitophagy; mitochondrial dysfunction; mitochondrial mutations; oxidative stress; SNPs	Review
Foreman et al. [5]	Redox Signaling and Sarcopenia: Searching for the Primary Suspect	aging; mitochondria; redox signaling; sarcopenia; skeletal muscle; peroxiredoxin	Review
Geary et al. [2]	Mitochondrial Functioning and the Relations among Health, Cognition, and Aging: Where Cell Biology Meets Cognitive Science	cognitive aging; dementia; cognitive ability; mitochondria; mitochondrial dysfunction; oxidative stress	Review
Lefranc et al. [3]	Adipocyte-Mineralocorticoid Receptor Alters Mitochondrial Quality Control Leading to Mitochondrial Dysfunction and Senescence of Visceral Adipose Tissue	mineralocorticoid receptor; mitochondrial dysfunction; adipose tissue; oxidative stress; senescence; metabolic syndrome	Article
Pagano et al. [6]	Aging-Related Disorders and Mitochondrial Dysfunction: A Critical Review for Prospect Mitoprotective Strategies Based on Mitochondrial Nutrient Mixtures	aging-related disorders; oxidative stress; mitochondria; mitochondrial dysfunction; mitochondrial nutrients; optic neuropathies; microbiome	Review

Overall, the five publications in this Special Issue demonstrate the wide variety of biological processes in which mitochondria are crucial players, which strengthens the importance of mitochondria in health and healthy ageing. We would like to thank all authors who contributed to the Special Issue. While many of the underlying mechanisms of mitochondrial dysfunction as a key factor in a variety of disorders need further work to be fully understood, this Special Issue highlights the interconnectedness of mitochondrial impacts and the growth of the field and future directions of this research.
